# Full genomic characterization of a porcine rotavirus strain detected in an asymptomatic piglet in Accra, Ghana

**DOI:** 10.1186/s12917-019-2226-9

**Published:** 2020-01-10

**Authors:** Samuel C. B. Stubbs, Osbourne Quaye, Maame Ekua Acquah, Samuel Mawuli Adadey, Iain R. L. Kean, Srishti Gupta, Barbara A. Blacklaws

**Affiliations:** 1 Department of Veterinary Medicine, University of Cambridge, Cambridge, UK; 2 West African Centre for Cell Biology of Infectious Pathogens (WACCBIP), Department of Biochemistry, Cell and Molecular Biology, University of Ghana, Volta Road, P. O. Box LG 54, Legon, Accra Ghana

**Keywords:** Rotavirus A, Full genome sequence constellation, Pig, Porcine, Ghana

## Abstract

**Background:**

The introduction of rotavirus A vaccination across the developing world has not proved to be as efficacious as first hoped. One cause of vaccine failure may be infection by zoonotic rotaviruses that are very variable antigenically from the vaccine strain. However, there is a lack of genomic information about the circulating rotavirus A strains in farm animals in the developing world that may be a source of infection for humans. We therefore screened farms close to Accra, Ghana for animals sub-clinically infected with rotavirus A and then sequenced the virus found in one of these samples.

**Results:**

6.1% of clinically normal cows and pigs tested were found to be Rotavirus A virus antigen positive in the faeces. A subset of these (33.3%) were also positive for virus RNA. The most consistently positive pig sample was taken forward for metagenomic sequencing. This gave full sequence for all open reading frames except segment 5 (NSP1), which is missing a single base at the 5′ end. The virus infecting this pig had genome constellation G5-P[7]-I5-R1-C1-M1-A8-N1-T7-E1-H1, a known porcine genotype constellation.

**Conclusions:**

Farm animals carry rotavirus A infection sub-clinically at low frequency. Although the rotavirus A genotype discovered here has a pig-like genome constellation, a number of the segments most closely resembled those isolated from humans in suspected cases of zoonotic transmission. Therefore, such viruses may be a source of variable gene segments for re-assortment with other viruses to cause vaccine breakdown. It is recommended that further human and pig strains are characterized in West Africa, to better understand this dynamic.

## Background

Group A rotaviruses (RVA) (family *Reoviridae*, genus *Rotavirus*) are the single most common etiology of acute gastroenteritis (AGE) among the young of both humans and domestic animals worldwide [1, 2]. The rotavirus genome is composed of 11 double-stranded RNA segments which encode six structural and five or six non-structural proteins. Currently, RVA strains are classified based on the open reading frame sequences of all the 11 genes as Gx-P[x]-Ix-Rx-Cx-Mx-Ax-Nx-Tx-Ex-Hx [3]. To date, at least 36 G-types, 51 P-types, 26 I-types, 22 R-types, 20 C-types, 20 M-types, 31 A-types, 22 N-types, 22 T-types, 27 E-types, and 22 H-types have been detected (https://rega.kuleuven.be/cev/viralmetagenomics/virus-classification/newgenotypes). Porcine RVA strains vary in the G and P types detected, but commonly have a I5-R1-C1-M1-A8-N1-T7-E1-H1 genetic backbone; although A1, T1 and occasional C2 genotypes have also been seen in porcine isolates [4].

The distribution of RVA strains found in humans and animals strongly supports host species barriers and restriction [5]. However, the detection of genotypes in humans that have been previously found in animals suggests that RVA is capable of undergoing adaptions permitting interspecies transmission [6, 7]. Animal and avian species have therefore become potential reservoirs of rotaviruses that can infect humans. Unusual rotavirus strains in humans are suspected to have evolved either as a single virus infection, or from genetic reassortment between human, animal and avian species as a result of co-infection [6, 7]. Therefore, there is a need to perform animal rotavirus surveillance and monitoring of the role of animal rotaviruses as a potential genetic reservoir for emerging rotavirus strains that are pathogenic to humans.

In Ghana, as in many developing countries, there is close contact between humans and domestic and/or farm animals. In various communities in developing countries, wild animals are also used as sources of meat/protein and thus come into direct contact with humans. These interactions could be prime causes of zoonotic transmission and result in possible genetic reassortment, ultimately leading to ineffective vaccination regimes. There are no sequence data for veterinary-associated RVA strains from Ghana apart from a limited number of incomplete sequences from bats. In this study, we report a full genome characterization of a porcine Group A rotavirus strain that was detected in an asymptomatic pig on a swine farm in Accra, Ghana. This genomic sequence contributes to the database of circulating rotavirus strains in sub-Saharan Africa.

## Results

### Detection of rotavirus positive samples

Of the normal faecal samples taken from 445 pigs and cattle, 27 were positive for rotavirus antigen using the Rotavirus Test Kit (of which 3 were from cattle [1.4% cattle samples], 24 from pigs [10.4% pig samples]), 6 of the 27 antigen-positive samples also tested positive for the presence of rotavirus RNA by real-time RT-PCR targeting the NSP3 gene, and a further 3 were positive for VP6 RNA using an RVA specific assay; only one of these samples was from cattle. Sample 14 from a pig gave the most consistent results with strong PCR products. The VP6 PCR product from this sample was Sanger sequenced and found to have the closest nucleotide sequence homology to an RVA I5 genotype strain by BLASTn. In order to further characterize the genome of the rotavirus present, this sample was subjected to metagenomic sequencing.

### Metagenomic sequencing

We next generated near complete sequence for all genomic segments of the RVA in sample (called strain below) 14 (Table 1). The strain 14 segments were genotyped using the RotaC v2.0 web tool [8] (Table 2), which gave a constellation of G5-P[7]-I5-R1-C1-M1-A8-N1-T7-E1-H1. This GX-P[X]-I5-R1-C1-M1-A8-N1-T7-E1-H1 genotype constellation was identical to previously described porcine RVA strains from Belgium, Thailand and Italy [4]. In particular, the genome segments I5, A8 and T7 are associated with porcine RVA strains (Table 2) [10, 15, 17, 18–24], but have also been detected in strains isolated from suspected cases of pig-human transmission, such as KisB332, BE2001, and NT0073. In comparison, human RVA strains from West Africa largely resembled the Wa-like reference genotype of GX-P[X]-I1-R1-C1-M1-A1-N1-T1-E1-H1, although one of these (Ghan-005), also had a DS-1-like I2 segment (Table 2) [25].

**Table 1:** RVA segment genotypes and closest homologues in GenBank

Protein	Genomic Segment (bp^a^)	Genome Coverage^b^	Genotype	Closest match in GenBank % nucleotide identity, name, [reference]	Reference	Percentage identity cut-off values between genotypes [3]
VP7	9 (1062)	1–1062	G5	96%, RVA/Human-wt/CHN/LL4260/2000/G5P[6]	[9]	80
				96%, RVA/Human-wt/CHN/LL3354/2000/G5P[6]	[9]	
VP4	4 (2359)	1–2355	P[7]	89%, RVA/Pig-tc/AUS/TFR-41/1986/G5P[9]	[12]	80
				89%, RVA/Pig-tc/KOR/174–1/2006/G8P[7]	[13]	
VP6	6 (1356)	1–1350	I5	98%, RVA/Pig-wt/CHN/DZ-1/2013/G5P[X]	ds	85
				96%, RVA/Pig-wt/CHN/WF-5-1/2014/G5P[X]	ds	
VP1	1 (3302)	1–3292	R1	94%, RVA/Human-wt/TWN/07-96 s1118/2007/G9P[19]	[10]	83
				93%, RVA/Pig-wt/TWN/2–3/2015/G9P[13]	[10]	
VP2	2 (2717)	1–2717	C1	94%, RVA/Pig-wt/CHN/JL01/2017/GXP[17]	ds	84
				88%, RVA/Human-wt/HUN/BP271/2000/G4P[6]	[11]	
VP3	3 (2591)	22–2574	M1	95%, RVA/Giant_Panda-tc/CHN/CH-1/2008/G1P[7]	[14]	81
				94%, RVA/Human-wt/CHN/LL3354/2000/G5P[6]	[9]	
NSP1	5 (1567)	13–1522	A8	98%, RVA/Human-tc/VNM/NT0042/2007/G4P[6]	[15]	79
				95%, RVA/Pig-wt/VNM/14226_39/2012/G4P[6]	[16]	
NSP2	8 (1059)	18–1046	N1	96%, RVA/Pig-wt/TWN/3–17/2015/G9P[23]	[10]	85
				96%, RVA/Human-wt/TWN/07-96 s1118/2007/G9P[19]	[10]	
NSP3	7 (1076)	17–1076	T7	95%, RVA/Human-wt/BEL/BE2001/2009/G9P[6]	[17]	85
				94%, RVA/Human-wt/COD/KisB332/2008/G4P[6]	[18]	
NSP4	10 (750)	16–741	E1	98%, RVA/Pig-wt/CHN/LLP48/2008/G9P[6]	ds	85
				98%, RVA/Pig-wt/CHN/HLJ/15/1/2015/G4P[23]	ds	
NSP5	11 (663)	12–639	H1	98%, RVA/Pig-wt/USA/OK.5.68/2008 ^c^	ds	91
				98%, RVA/Pig-wt/CHN/NMTL/2008/G9P[23]	[19]	

*ds* direct submission

^a^Reference sequence length

^b^Sequences were aligned against the reference genotypes to determine segment coverage

^c^Strain OK.5.68 is OK.5.68a G5P[6] or OK.5.68b G9P[7] but the NSP5 is unassigned to a or b

**Table 2:**
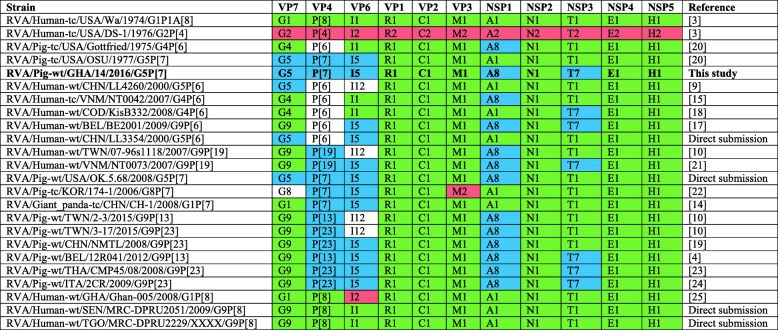
Genotype constellations of RVA strain 14, reference sequences, and the most homologous strains in GenBank

The Ghanaian porcine RVA strain 14 (RVA/Pig-wt/GHA/14/2016/G5P[7]) genotype constellation is shown in bold in comparison to reference RVA human strains, Wa and DS-1, and porcine strains, Gottfried and OSU; strains that were fully characterised in GenBank with the top BLASTn hits for each strain 14 segment; three typical porcine strains of the GX-P[X]-I5-R1-C1-M1-A8-N1-T7-E1-H1 constellation; and three human strains from West Africa. Green – Wa-like genogroup; pink –DS-1-like genogroup; blue – typical porcine genotype [4]

BLASTn analysis of the individual segments revealed 89–98% nucleotide similarity to the closest existing sequences in the GenBank nucleotide database (Table 1). The sequences with the greatest similarity to strain 14 segments included isolates from both pigs and humans, and the majority of these sequences (17/22) originated from countries in Asia. For 4 of the segments (VP4, VP6, NSP4 and NSP5), the matches with the greatest identity were exclusively of porcine origin. The VP2, VP3, NSP1 and NSP2 segments displayed similarity to a mixture of porcine, human and giant panda strains. The VP7, VP1, NSP1, and NSP3 segments most closely resembled strains that had been isolated from humans, originating from China, Taiwan, Vietnam and Belgium.

### Phylogenetic analysis

**Fig. 1:**
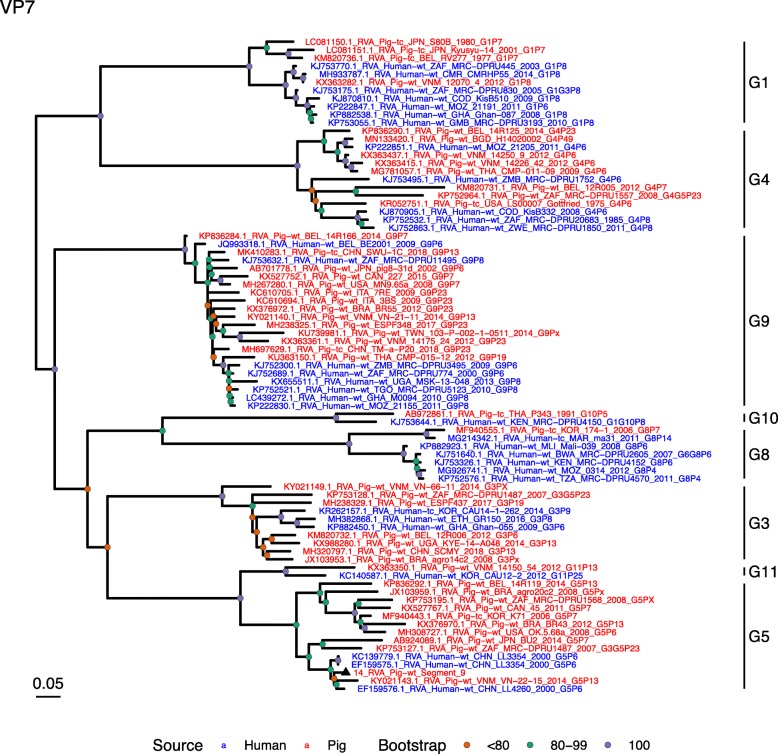
Maximum Likelihood Phylogeny of VP7, genomic segment 9. The tree includes global pig RVA strains and African strains of human origin retrieved from GenBank. We also included the three most closely related RVA strain segments, as identified by BLASTn, regardless of origin. Clades containing large clusters of closely related or identical sequences were pruned to show representative viral strains

**Fig. 2:**
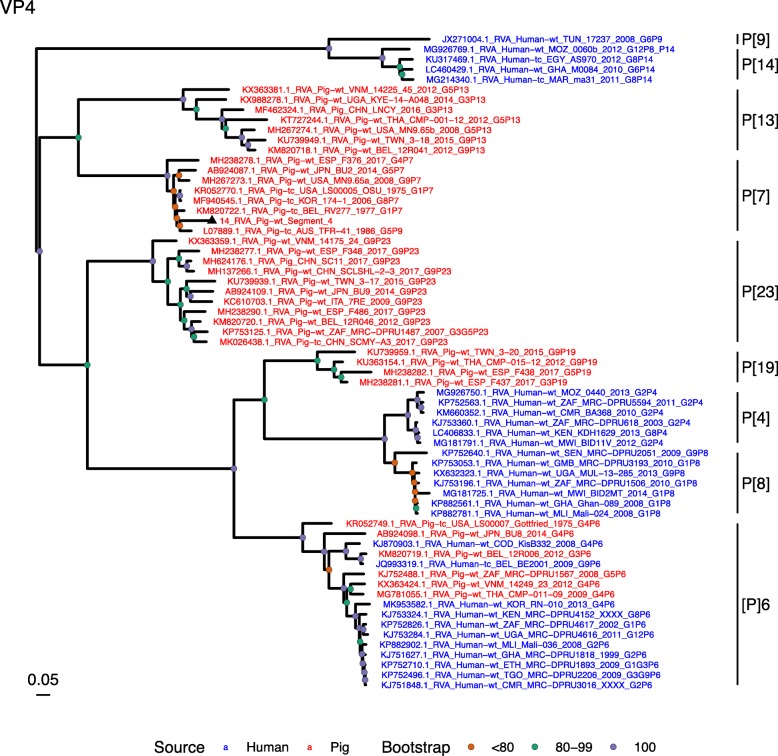
Maximum Likelihood Phylogeny of VP4, genomic segment 4. The tree includes global pig RVA strains and African strains of human origin retrieved from GenBank. We also included the three most closely related RVA strain segments, as identified by BLASTn, regardless of origin. Clades containing large clusters of closely related or identical sequences were pruned to show representative viral strains

**Fig. 3:**
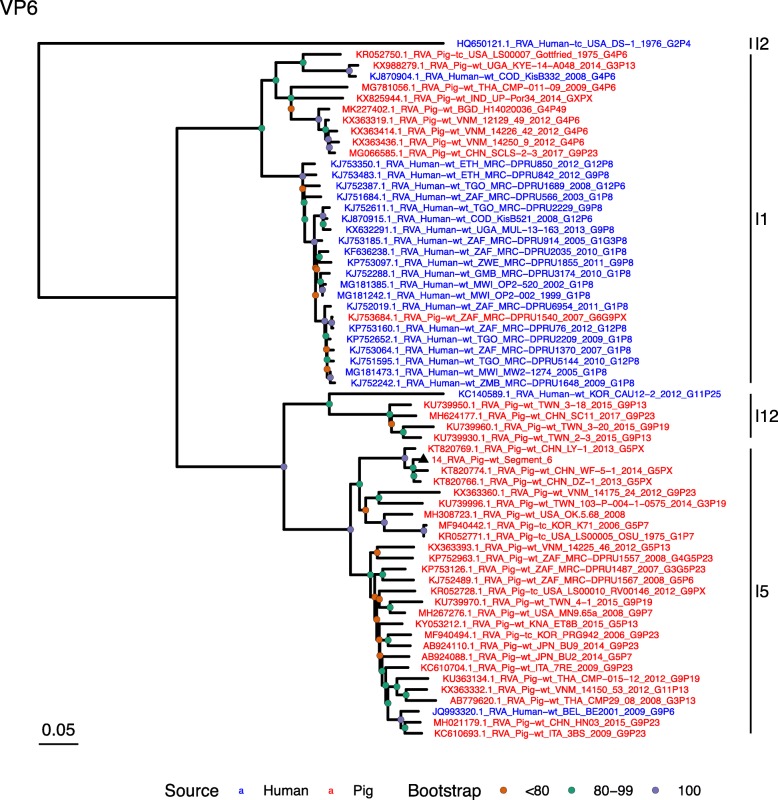
Maximum Likelihood Phylogeny of VP6, genomic segment 6. The tree includes global pig RVA strains and African strains of human origin retrieved from GenBank. We also included the three most closely related RVA strain segments, as identified by BLASTn, regardless of origin. Clades containing large clusters of closely related or identical sequences were pruned to show representative viral strains

**Fig. 4:**
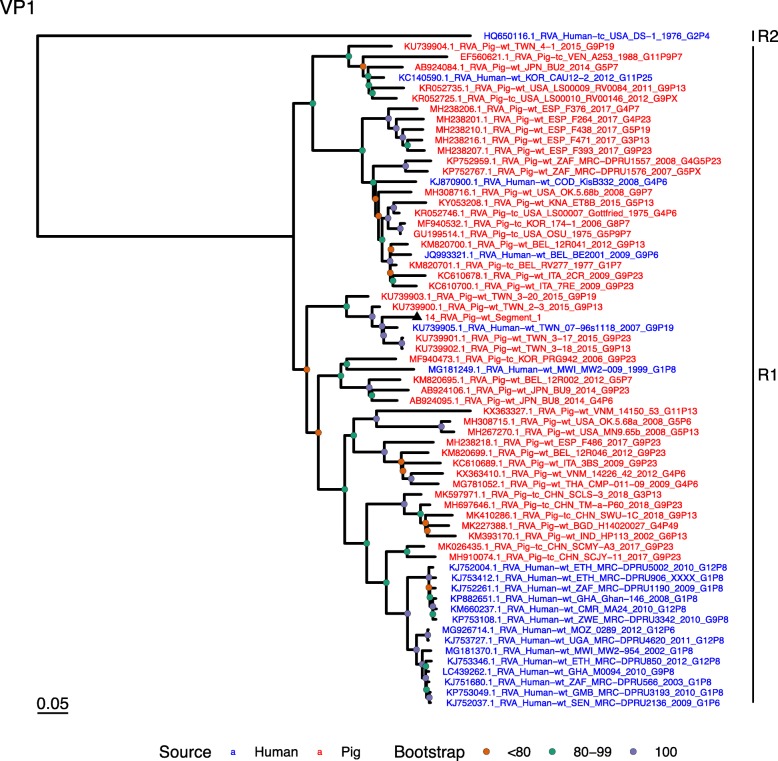
Maximum Likelihood Phylogeny of VP1, genomic segment 1. The tree includes global pig RVA strains and African strains of human origin retrieved from GenBank. We also included the three most closely related RVA strain segments, as identified by BLASTn, regardless of origin. Clades containing large clusters of closely related or identical sequences were pruned to show representative viral strains

**Fig. 5:**
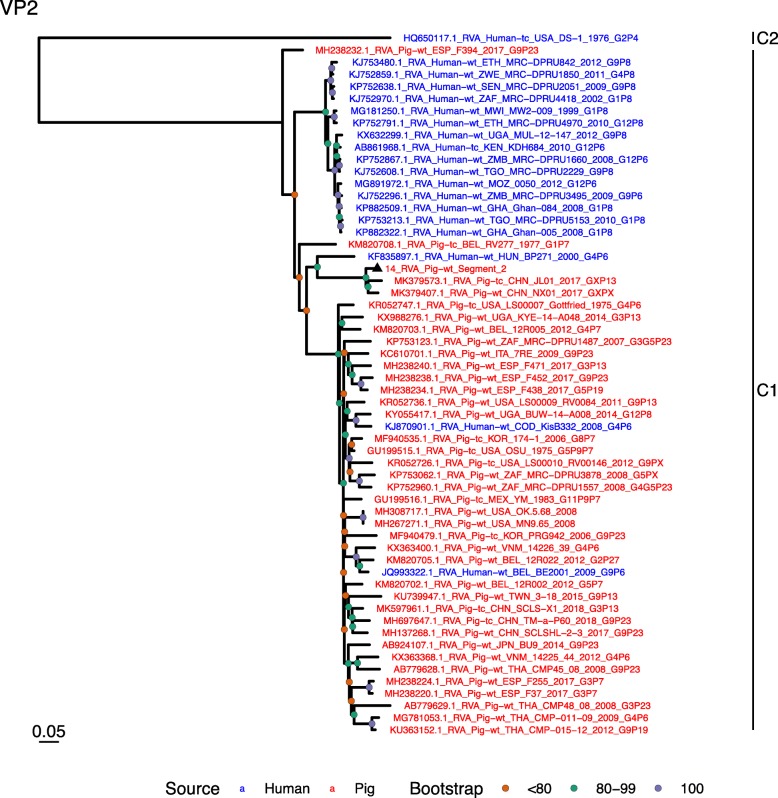
Maximum Likelihood Phylogeny of VP2, genomic segment 2. The tree includes global pig RVA strains and African strains of human origin retrieved from GenBank. We also included the three most closely related RVA strain segments, as identified by BLASTn, regardless of origin. Clades containing large clusters of closely related or identical sequences were pruned to show representative viral strains

**Fig. 6:**
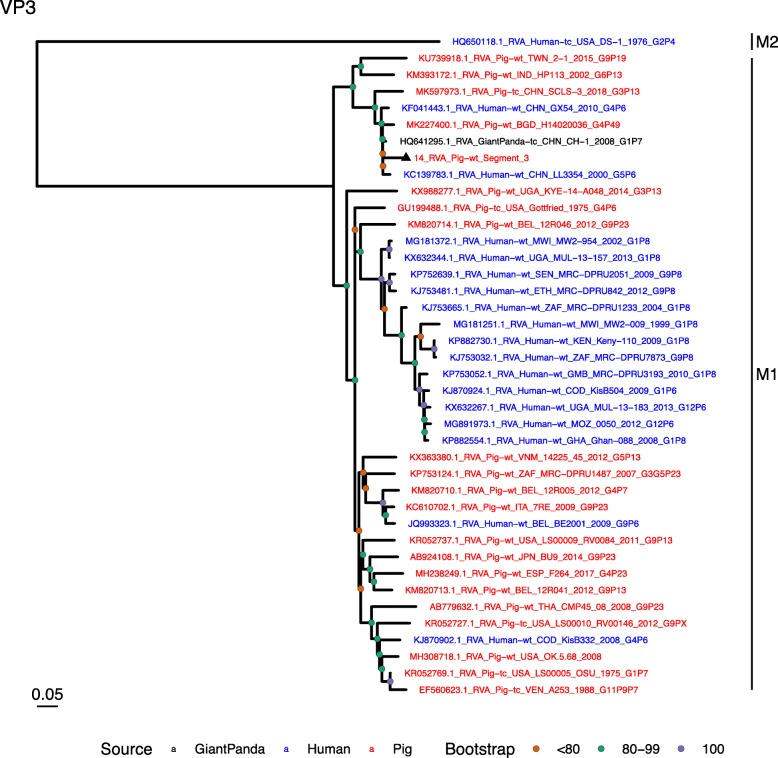
Maximum Likelihood Phylogeny of VP3, genomic segment 3. The tree includes global pig RVA strains and African strains of human origin retrieved from GenBank. We also included the three most closely related RVA strain segments, as identified by BLASTn, regardless of origin. Clades containing large clusters of closely related or identical sequences were pruned to show representative viral strains

**Fig. 7:**
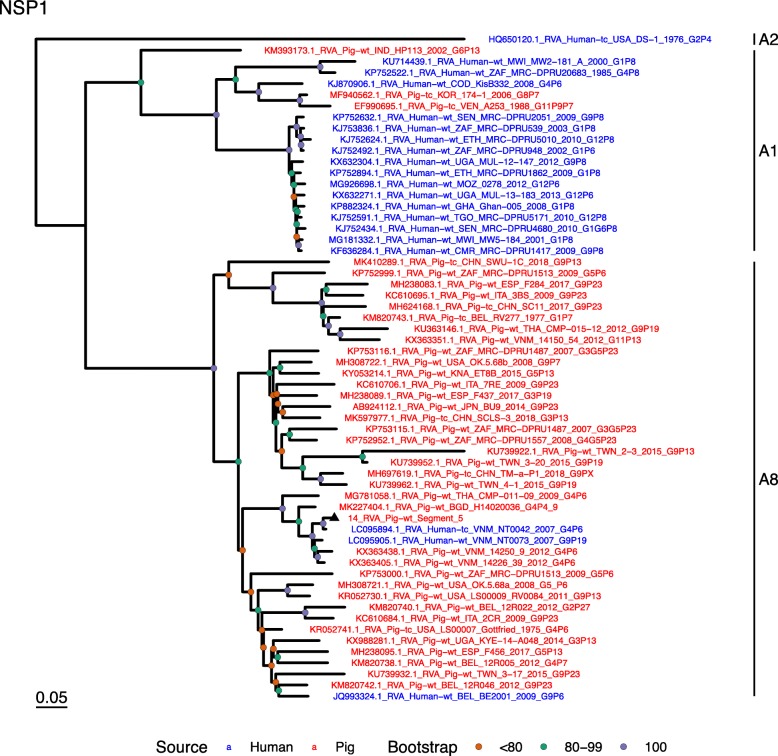
Maximum Likelihood Phylogeny of NSP1, genomic segment 5. The tree includes global pig RVA strains and African strains of human origin retrieved from GenBank. We also included the three most closely related RVA strain segments, as identified by BLASTn, regardless of origin. Clades containing large clusters of closely related or identical sequences were pruned to show representative viral strains

**Fig. 8:**
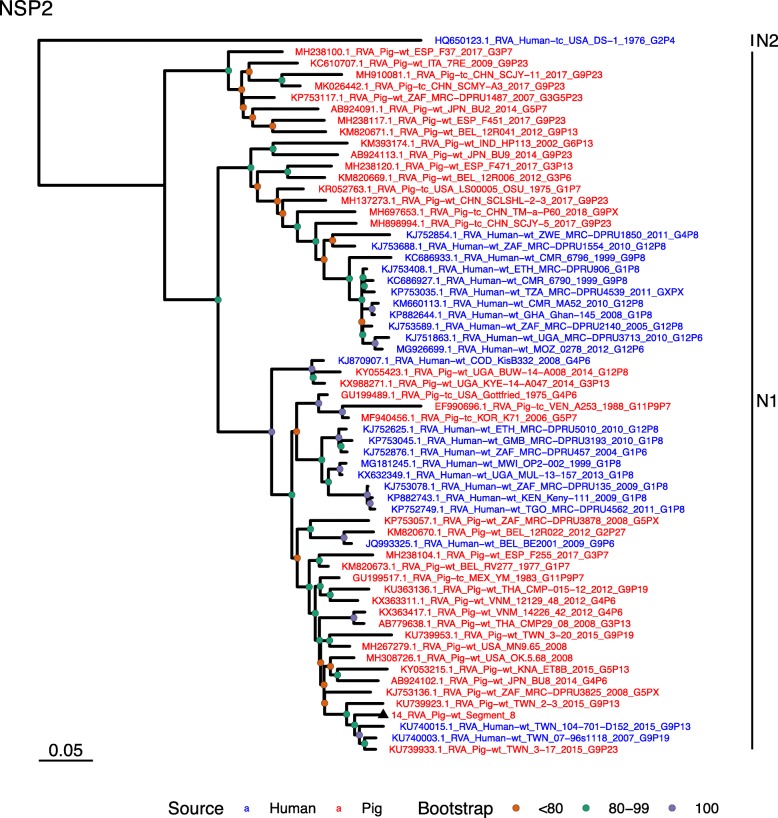
Maximum Likelihood Phylogeny of NSP2, genomic segment 8. The tree includes global pig RVA strains and African strains of human origin retrieved from GenBank. We also included the three most closely related RVA strain segments, as identified by BLASTn, regardless of origin. Clades containing large clusters of closely related or identical sequences were pruned to show representative viral strains

**Fig. 9:**
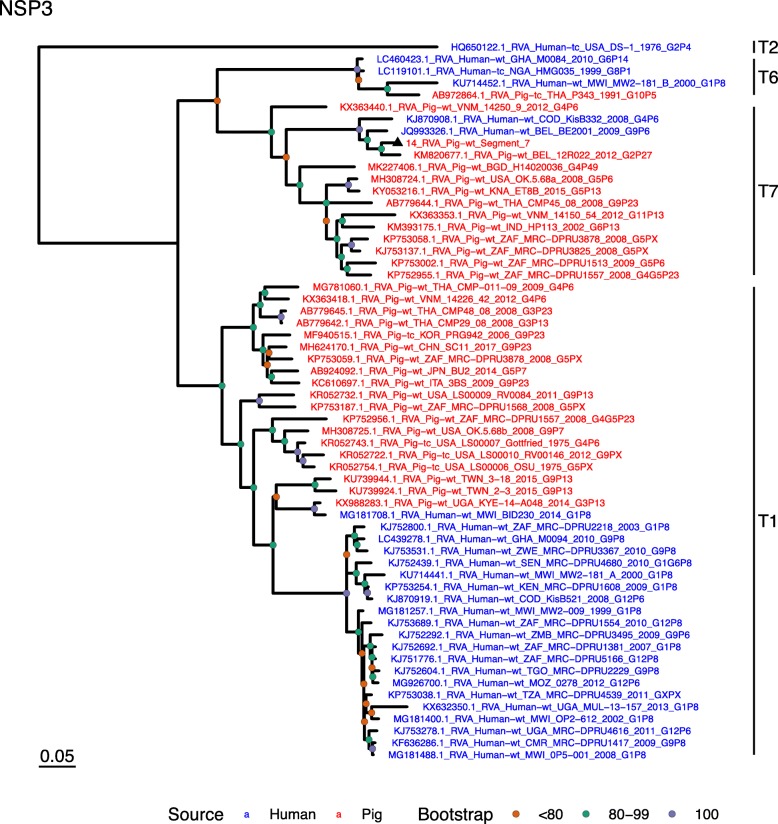
Maximum Likelihood Phylogeny of NSP3, genomic segment 7. The tree includes global pig RVA strains and African strains of human origin retrieved from GenBank. We also included the three most closely related RVA strain segments, as identified by BLASTn, regardless of origin. Clades containing large clusters of closely related or identical sequences were pruned to show representative viral strains

**Fig. 10:**
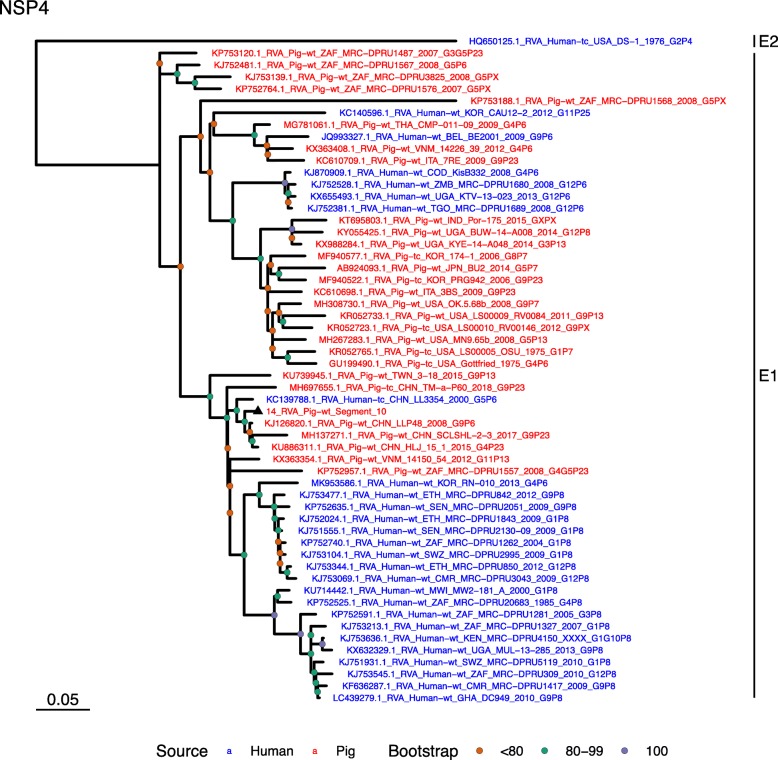
Maximum Likelihood Phylogeny of NSP4, genomic segment 10. The tree includes global pig RVA strains and African strains of human origin retrieved from GenBank. We also included the three most closely related RVA strain segments, as identified by BLASTn, regardless of origin. Clades containing large clusters of closely related or identical sequences were pruned to show representative viral strains

**Fig. 11:**
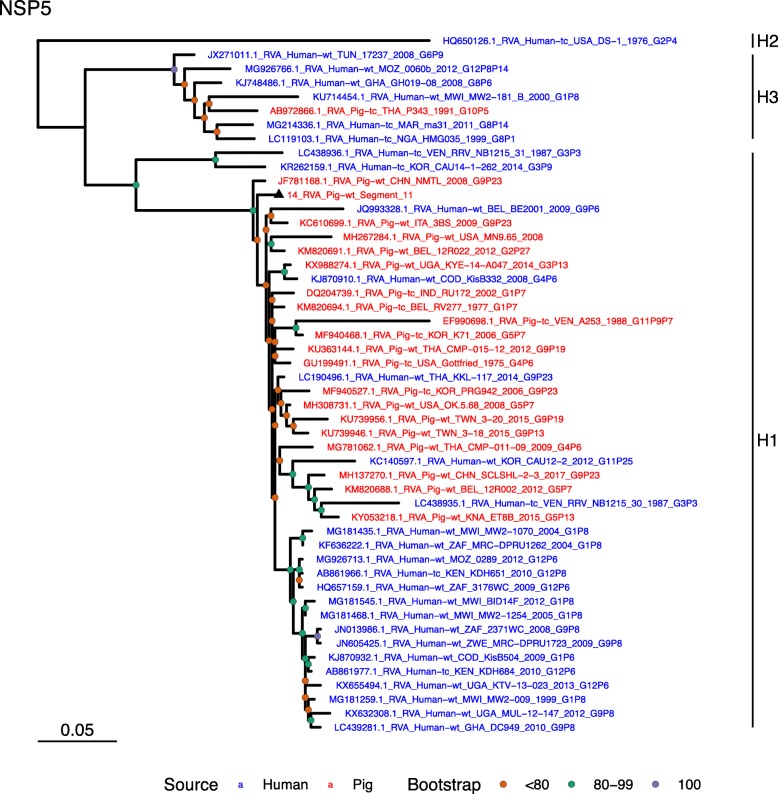
Maximum Likelihood Phylogeny of NSP5, genomic segment 11. The tree includes global pig RVA strains and African strains of human origin retrieved from GenBank. We also included the three most closely related RVA strain segments, as identified by BLASTn, regardless of origin. Clades containing large clusters of closely related or identical sequences were pruned to show representative viral strains

Phylogenetic analysis of each genomic segment was performed against porcine RVA strains and African strains of human origin retrieved from GenBank. We also included the three most closely related RVA strain segments, as identified by BLASTn, regardless of origin. Clades containing large clusters of closely related or identical sequences were pruned to show representative viral strains.

(i.) **Outer capsid proteins VP7 and VP4**: Segment 9 (VP7) of strain 14 clustered within the genotype G5 clade, which predominantly consisted of strains of porcine origin (Fig. 1). However, the strain 14 segment formed part of a smaller clade within this genotype, which contained sequences derived from both pig and human. The closest related sequences to the strain 14 segment were two human RVA strains isolated from patients in China in 2000 [9] (Table 1). However, the predominance of porcine strains in this clade suggests that these human infections were of zoonotic origin.
  The nucleotide sequence of strain 14 segment 4 (VP4) was most homologous to two porcine strains from Australia and Korea isolated in 1986 and 2006 respectively (Table 1) [12, 13], although shared identity was only 89%, suggesting these strains were not close relatives. Strain 14 VP4 sits within the P[7] clade which is derived from only pig isolates from a variety of global sources (Fig. 2) suggesting it is a segment with a porcine lineage.
(ii.) **Inner capsid protein (VP6):** Segment 6 (VP6) of strain 14 clustered within the I5 clade, but formed a small, distinct outgroup with three Chinese isolates from 2013/14 (Fig. 3). The Chinese strains were all isolated by the same group (direct submissions to GenBank) so are likely to be from the same region. The I5 clade is dominated by porcine isolates as expected [4] with one human isolate shown, suggesting a porcine lineage for this genomic segment in strain 14.
(iii.) **Viral replication enzymes (VP1 and VP3) and core scaffold protein (VP2):** Strain 14 genomic segments 1, 2 and 3 all clustered within genotype 1 (Wa-like) of the corresponding genes (Figs. 4, 5, 6). For VP1, the most homologous sequences came from a human and a pig in Taiwan, isolated in 2007 and 2015 respectively (Table 1). These Taiwanese strains were sequenced as part of the same study, which concluded that the similarity of the human strain to those detected in pigs was suggestive of zoonotic origin [10]. This conclusion is supported by our phylogenetic analysis, which shows a separate R1 clade containing the human Wa-like isolates (Fig. 4). Similar to VP1, phylogenetic analysis of VP2 produced 2 major sub-clusters within the C1 clade: one containing only human strains and one predominantly containing pig strains (Fig. 5). The VP2 segment of strain 14 formed part of a small outgroup within the latter clade, suggesting that this segment originates from a pig lineage. The outgroup contained two closely related Chinese strains from 2017 (94% nucleotide identity), and a more distantly related human-derived strain from a pediatric patient in Hungary (88% nucleotide identity), thought to be a further case of pig-human transmission, due to its similarity to known pig strains [11]. The VP3 segment M1 clade again showed 2 major sub-clusters separating strains of pig and human origin (Fig. 6). However, the strain 14 VP3 segment formed part of a smaller clade. This clade predominantly contained porcine strains, suggesting a porcine ancestral origin, but the strain 14 segment itself was most similar to a 2008 strain from a giant panda and two human strains (2000 and 2010), all from China (Table 1) [9, 14]. Nucleotide similarities of 94–95% to strain 14 suggest that cross-species transmission is likely to have occurred.
(iv.) **Non-structural proteins (NSP1–5/6):** The interferon antagonist non-structural protein 1, NSP1, of strain 14 was most closely related to a group of human and porcine strains from Vietnam (Table 1) [15, 16]. On phylogenetic analysis, the segment clustered with the A8 clade. This clade constituted porcine isolates with only occasional human isolates (Fig. 7). The ancestral origin of this strain 14 segment is therefore suggested to be porcine as these are the dominant isolates in its cluster. The NSP2 (viroplasm-associated protein) segment 8 of strain 14 was most similar to a 2015 porcine isolate and a 2007 human isolate both from Taiwan (Table 1) [10]. These cluster in the N1 clade which had 3 major sub-clusters (Fig. 8). The first contained only porcine isolates. The second and third sub-clusters came from a common branch and within each there are further sub-groups that show either pig or human specificity. Segment 8 of strain 14 clustered in a minor sub-group that contained both porcine and human isolates which is within a porcine isolate dominated sub-cluster suggesting the ancestral origin of this segment was from pig. Segment 7 NSP3 (translation enhancer protein) of strain 14 was most homologous at the nucleotide level to two strains of human origin, a 2009 isolate from Belgium and a 2008 isolate from Côte d’Ivoire (Table 1) [17, 18]. These form a distinct cluster within the T7 genotype clade, which is largely porcine-dominated (Fig. 9). It is hard to definitely suggest a likely origin of the strain 14 segment 7 due to the even mix of porcine and human strains. However, outside of this cluster, the T7 genotype contains only pig strains, and so a porcine origin is most-likely. The viral enterotoxin (NSP4) genomic segment 10 was most homologous at the nucleotide level with two pig isolates from China, one from 2008 and one from 2015 (Table 1). These cluster in the E1 clade (Fig. 10). The E1 clade is broadly separated into sub-clusters of either human or porcine origin with only 2 small sub-groups showing mixed origin. Strain 14’s segment 10 was in a pig dominated sub-group although there was a closely related 2000 human isolate LL3354 from China also within this group. This would suggest that this genomic segment is of porcine origin. The viroplasm-associated non-structural protein, NSP5, from segment 11 was most similar to two 2008 pig isolates; one from USA and one from China (Table 1) [19]. The maximum-likelihood phylogenetic tree of segment 11 showed NSP5 to sit in the H1 clade which had two major sub-clusters; one was human and the other was mixed human and porcine isolates (Fig. 11). The second sub-cluster further differentiated into a human only, a porcine only and a mixed sub-group. Strain 14 was within the mixed sub-group. The majority of strains within this group were porcine, suggesting that this genomic segment was also of a porcine lineage.

## Discussion

We report here the near complete genome of an RVA isolate detected in the faeces of an asymptomatic pig in Ghana. We propose to call this strain RVA/pig-wt/GHA/14/2016/G5P[7]. The genomic constellation was G5-P[7]-I5-R1-C1-M1-A8-N1-T7-E1-H1, which is similar to prototype porcine RVA strains, in particular to Ugandan isolates sequenced by Amimo et al. (2015) [26] which were G5P[13] and G2, G9 and G11 Canadian strains sequenced by Martel-Paradis et al. (2013) [27] and the porcine lineage genotype constellation described by Theuns et al. (2015) [4] (GX-P[X]-I5-R1-C1-M1-A8-N1-T7-E1-H1).

Despite being of a porcine lineage, several segments of strain 14 were found to be most closely related to strains derived from humans. However, phylogenetic analysis suggests that these human strains are more likely to be porcine-like, rather than strain 14 being human-like. Regardless, this similarity between a porcine RVA strain and several human strains provides further evidence that transmission of such porcine RVA strains to humans can occur, and a paucity of RVA sequence data from West Africa makes it difficult to determine whether or not zoonotic cross-species transmission is common in the region. This is important as zoonotic infection of humans by RVA from animals or the re-assortment of virus segments between human and animal rotaviruses to cause antigenic shift and/or increase virulence are considered factors that may affect the effectiveness of rotavirus vaccine campaigns, especially in developing countries where there is documented evidence of mixed infections [28].

Ghana introduced the vaccine Rotarix, a Wa-1-like vaccine (G1-P[8]-I1-R1-C1-M1-A1-N1-T1-E1-H1), in May 2012. The predominant circulating RVA strains in hospitalized paediatric cases has varied since its introduction [29]. However, Lartey et al. (2018) [29] only characterized the VP4 and VP7 encoding genomic segments in their study. It is therefore difficult to compare the full genome we report here to post-vaccine human samples; although the VP4 and VP7 genotypes of strain 14 are different to those of the local human strains described by Lartey et al. (2018) [29].

It is interesting to note that the pigs and cattle on the farms tested showed no evidence of clinical gastroenteric disease when the samples were taken. Furthermore, the metagenomic sequencing, in addition to sequence reads of RVA, also revealed the presence of other eukaryotic RNA viruses associated with both sub-clinical and clinical gastroenteric infection. This demonstrates that healthy pigs are viral carriers and therefore potential sources of infection for other animals. Indeed, in a study analyzing young pig faeces for sub-clinical infection with RVA in East Africa, 26% of animals were positive for RVA, with a range of 79% in nursing piglets reducing to 6% in grower piglets [26]; we were within this range with 10.4% of the animals sampled (all under 1 year old) in Ghana being positive. Amimo et al. (2015) [26] also showed that infection prevalence varied with pig density, management system and age.

There is a porcine rotavirus vaccine available (Prosystem Rota from Merck Animal Health) which is based on modified porcine RVA strains Gottfried (G4P[6]) and OSU (G5P[7]). This is not known to be used in Ghana. The sequence obtained above is 87% identical to OSU G5 VP7 whilst it is 96% identical to human strains (Table 1). This suggests that the strain sequenced here is not vaccine derived and that this is not the reason for its apparent lack of virulence in the sampled pig.

The variation and unusual strains detected by Lartey et al. (2018) [29] in humans suggest selection pressure is occurring by vaccination. This may provide niches for infection by zoonotic RVA strains. The genomic constellation detected in the sub-clinically infected pig in this study contained segment genotypes that have been detected in human infections previously [17, 30, 31]. Sub-clinically infected farm animals are therefore a potential source of virus for infection of humans, with or without reassortment of the different genomic segments.

Finally, it is interesting to note that the isolates with the greatest similarity to strain 14 were predominantly of Asian origin. Whilst it is possible that Asian and West African strains are highly related, it is more likely that this outcome is due to a limited number of porcine RVA sequences from West Africa, demonstrating the importance of this first sequence from an agriculturally important species in Ghana.

## Conclusions

Pigs and cattle carry RVA infection sub-clinically at low frequency. It is possible to sequence the virus in these samples and determine full genome constellation data using a metagenomic approach. The RVA genotype discovered has a porcine-like genome constellation. However, a number of the segments most closely resembled those isolated from humans in suspected cases of zoonotic transmission. Therefore, such viruses may be a source of variable gene segments for re-assortment with other viruses to cause vaccine breakdown. It is recommended that further human and pig strains are characterized in West Africa, to better understand this dynamic.

## Methods

### Aim and design of the study

The study aimed to determine the prevalence and strains of subclinical carriage of rotavirus A in farm animals in Ghana as these are a possible source of infection of humans. Swine and cattle farms located in the northeastern part of Accra were randomly selected for collection of stool samples from clinically normal animals. The samples were collected from animals under 1 year old under aseptic conditions and either stored at − 20 ^o^ C or used to prepare 10% fecal suspensions in phosphate-buffered saline solution. The suspensions were vortexed, centrifuged at 600 x ***g*** for 10 min, and the supernatant used in an immunochromatographic assay for the detection of rotavirus antigen and for total RNA extraction. The immunochromatographic assay was performed using the ProFlow Rotavirus test kit following the manufacturer’s manual.

Total RNA was extracted from the samples that were positive for rotavirus antigen using the GenElute extraction kit (Sigma Aldrich) with some modifications. Briefly, 350 μL of 10% stool suspension supernatant were mixed with an equal volume of GenElute lysis buffer, 350 μL 70% ethanol added and vortexed. The mixture was spun through a GenElute filtration column. 750 μL of Trizol was added to the filtrate, vortexed, and incubated at room temperature for 5 min. Chloroform (200 μL) was added to the mixture, vortexed, and incubated at room temperature for 3 min. The mixture was centrifuged at 300 x ***g*** for 5 min and 250 uL of the clear upper phase was pipetted off and mixed with 250 μL of 70% ethanol. The mixture was transferred to a GenElute binding column and the normal RNA purification method then followed. Two lots of 30 μL elution solution were used, collecting both eluates in the same tube for a higher RNA yield. The concentration and purity of the extracted RNA was determined by the NanoDrop spectrophotometer.

### Reverse transcriptase-PCR

Sample RNA was first tested for the presence of rotavirus using a one-step NSP3 RT-PCR [32]. This was performed using the Kapa Probe Fast Universal One-Step qRT-PCR kit and the manufacturer’s conditions with RNA diluted 1/5 and the primers (NSP3-F, NSP3-R) and probe (NSP3-p) from Zeng et al. (2008) [32] (Table 3). The reaction conditions were 42 °C for 5 min, 95 °C for 5 min, then 40 cycles of 95 °C for 5 s, 60 °C for 30 s and these were performed in a Rotorgene 600 (Qiagen). The RNA was then tested for the presence of genomic segment 6 (VP6) using the rotavirus group A specific primers VP6F and VP6R from Iturriza-Gomara et al. (2002) [33] (Table 3). Reverse transcription was first carried out using Superscript III (ThermoFisher Scientific) and random hexamer primers as per the manufacturer’s instructions, RNAse H treated, then cDNA was diluted 1/5 and taken into a PCR with Platinum Pfx (94 °C for 5 min, 35 cycles of 94 °C for 15 s, 55 °C for 30 s, 68 °C for 30 s with a final extension of 68 °C for 10 min). This reaction gave a predicted band size of 380 bp which was detected on a 2% agarose gel. If present, the band was purified from the gel and sequenced using the Sanger sequencing method and VP6F as primer.

**Table 3:** Primers and probes used in RT-PCR

Genome Segment	Protein	Primer/probe sequence	Predicted product size (bp)	Reference
7	NSP3	NSP3-F: ACCATCTWCACRTRACCCTCTATGAG NSP3-R: GGTCACATAACGCCCCTATAGC NSP3-p: FAM5’-AGTTAAAAGCTAACACTGTCAAA-3’MGB	87 bp	[32]
6	VP6	VP6F: GACGGVGCRACTACATGGT VP6R: GTCCAATTCATNCCTGGTGG	380 bp	[33]
2	VP2	VP2F1: ACAAGCGGCAAATGACTGTT VP2R1: GGTTGATGACTTGTCCACTCC	398 bp	This paper
11	NSP5	NSP5F1: ACAACGTCAACTCTTTCTGGA NSP5R1: TGCTTGAAGGTCGTGATTGC	232 bp	This paper
	SISPA	FR26RV-N: GCCGGAGCTCTGCAGATATCNNNNNN FR20RV: GCCGGAGCTCTGCAGATATC		[34]

### Metagenomic sequencing

#### Illumina library preparation and sequencing

RNA was reverse transcribed and amplified following a sequence-independent single-primer amplification and reverse transcription (SISPA-RT) protocol as previously described [34]. Briefly, 5 μL of RNA was combined with 1 μl of 10 μM primer FR26RV-N (Table 3), 1 μL of 10 mM dNTPs, and 6 μl of RNase-free water. The mixture was heated to 65 °C for 5 min and placed on ice before adding a mastermix consisting of: 1 μl (40 U) of RNase OUT (Thermo Fisher Scientific); 1 μl (200 U) of Superscript III reverse-transcriptase (Thermo Fisher Scientific); 4 μl of 5X Superscript III reaction buffer; and 1 μL of 0·1 M dithiothreitol, to give a final volume of 20 μL. The mixture was incubated in a thermocycler at 25 °C for 5 min, followed by 50 min at 50 °C. The reaction was terminated by heating to 94 °C for 3 min, and then cooled to 4 °C. Second-strand cDNA synthesis was performed by adding 0.5 μl (2·5 U) of large Klenow fragment (New England Biolabs) to each reaction and incubating at 37 °C for 60 min followed by 75 °C for 10 min to inactivate the enzyme.

SISPA amplification was performed in duplicate for each sample, using the cDNA reaction mixture as direct input. The final reaction mixture consisted of 5 μl of cDNA, 2 μl of 10 μM primer FR20RV (Table 3), 1 μl of 10 mM dNTP mixture, 0.5 μl of Phusion DNA polymerase (Thermo Fisher Scientific), 10 μl of 5 X Phusion high-fidelity reaction buffer and 31.5 μl of nuclease-free water. The PCR reaction was performed by heating to 98 °C for 30 s followed by 14 cycles of 98 °C for 10 s, 62 °C for 30 s, 72 °C for 120 s followed by a final extension at 72 °C for 10 min. Duplicate reactions were combined and the amplified DNA was purified using KAPA PureBeads (Roche) at a 1:1 ratio, eluting in 20 μl of buffer EB (Qiagen).

DNA was fragmented to an average length of 400 bp by sonication (microTUBEs, Covaris) and indexed Illumina sequencing libraries were prepared from the fragmented DNA using the NEBNext Ultra II DNA library preparation kit (NEB). 2 × 150 bp paired-end reads were generated on the Illumina MiSeq using v3 reagent chemistry, producing approximately 2.5 × 10^6^ reads per sample.

### Sequence data analysis

Sequencing data was de-multiplexed and imported to the CLC Genomics Workbench software (Qiagen) for analysis. The reads were trimmed to remove low-complexity and low-quality regions together with the SISPA primer sequence. The trimmed reads were then mapped to a custom database containing the reference genomes of 1377 vertebrate viruses taken from the NCBI RefSeq database [35]. To account for strain variability, the mapping parameters allowed for up to 36% mismatch between reads and the reference sequence (80% identity over 80% of the read). Majority consensus sequences generated by mapping were extracted and confirmed by alignment to the complete nucleotide database using the NCBI BLASTn tool [36]. The sequence with the best match was downloaded and used as a reference for a second round of mapping in order to generate the most complete alignment possible. Primer3 software [37] was used to generate PCR primers based on the consensus sequence of any incomplete segments. PCR products were amplified using Phusion DNA polymerase and sequenced by Sanger sequencing to fill gaps that could not be completed using the metagenomic data alone. The resulting Sanger-generated sequences were then aligned to their incomplete consensus sequence and an updated consensus was called. Rotavirus consensus sequences were extracted from the alignment and genotyped using the RotaC classification tool (http://rotac.regatools.be). Phylogenies were created with sample 14 sequences and the most homologous genotypes identified by BLASTn in GenBank after removal of duplicate sequences from the same strains. Nucleotide alignments were carried out using ClustalOmega (https://www.ebi.ac.uk/Tools/msa/clustalo/) then refined in SeaView v4 [38] using amino acid homologies. Phylogenetic trees were reconstructed using maximum likelihood in IQ-TREE v1.6.11 [39] using model selection [40], a more thorough nearest neighbour interchange search (‘-allnni’), and 1000 iterations of the ultrafast bootstrap approximation (−bb 1000) [41]. The resulting trees were visualised using ggtree [42] in R v3.5.1 [43].

## Data Availability

Accession numbers in GenBank for the sample 14 rotavirus sequences are: MN102365-MN102375.

## Abbreviations

BLASTn: Basic local alignment search tool for nucleotides
cDNA: Copy DNA
DNA: Deoxyribonucleic acid
NCBI: National Center for Biotechnology Information
NSP: Non-structural protein
PCR: Polymerase chain reaction
qRT-PCR: Quantitative reverse transcription polymerase chain reaction
RefSeq: Reference sequence
RNA: Ribonucleic acid
RT-PCR: Reverse transcription polymerase chain reaction
RVA: Group A rotavirus
SISPA-RT: Sequence-independent single-primer amplification and reverse transcription
VP: Virion protein
